# von Willebrand Factor and ADAMTS13 Predict Therapeutic Efficacy and Severe Adverse Events in Advanced Hepatocellular Carcinoma Treated With Atezolizumab–Bevacizumab

**DOI:** 10.1002/jgh3.70484

**Published:** 2026-10-27

**Authors:** Naoki Nishimura, Hiroaki Takaya, Tadashi Namisaki, Yusuke Komeda, Satoshi Iwai, Akihiko Shibamoto, Yuki Tsuji, Yukihisa Fujinaga, Koh Kitagawa, Shinya Sato, Norihisa Nishimura, Kosuke Kaji, Hitoshi Yoshiji

**Affiliations:** ^1^ Department of Gastroenterology Nara Medical University Nara Japan

**Keywords:** angiogenesis, bevacizumab, biomarkers, liver neoplasms, thrombosis

## Abstract

**Aims:**

Atezolizumab combined with bevacizumab (AB therapy) is a standard first‐line treatment for advanced hepatocellular carcinoma (HCC). However, clinically useful biomarkers for predicting therapeutic efficacy and severe adverse events (AEs) remain limited. Beyond its role in thrombosis, ADAMTS13–von Willebrand factor (VWF) axis is increasingly recognized for its involvement in angiogenesis and immune modulation. This study aimed to investigate the clinical significance of the ADAMTS13–VWF axis in patients receiving AB therapy.

**Methods:**

A total of 58 patients with advanced HCC treated with AB therapy were enrolled. Plasma VWF antigen (VWF:Ag) and ADAMTS13 activity (ADAMTS13:AC) were measured before treatment initiation. Therapeutic efficacy was assessed using Response Evaluation Criteria in Solid Tumors (RECIST) version 1.1 and modified RECIST (mRECIST), while safety was assessed according to Common Terminology Criteria for AEs version 5.0.

**Results:**

Multivariate analyses identified lower baseline VWF:Ag (< 180%) and ADAMTS13:AC (≤ 60%) as independent predictors of superior therapeutic efficacy according to both RECIST and mRECIST criteria. Patients with low VWF:Ag (< 180%) and ADAMTS13:AC (≤ 60%) exhibited significantly prolonged overall survival. Lower baseline VWF:Ag to ADAMTS13:AC (VWF/ADAMTS13) ratios (< 3) were independently associated with severe AEs (Grade ≥ 3). Plasma VWF:Ag and ADAMTS13:AC levels did not significantly change after AB therapy.

**Conclusion:**

Baseline VWF:Ag and ADAMTS13:AC independently predict therapeutic efficacy, whereas the baseline VWF/ADAMTS13 ratio independently predicts severe AEs in patients with HCC receiving AB therapy. These biomarkers may help optimize patient selection and manage treatment‐related toxicities by reflecting tumor angiogenic activity and host immune status.

## 1 Introduction

Hepatocellular carcinoma (HCC) is one of the most prevalent malignancies worldwide and a leading cause of cancer‐related mortality [1, 2]. Atezolizumab in combination with bevacizumab (AB therapy) has been recently established as standard first‐line systemic treatment for unresectable HCC, demonstrating superior overall survival (OS) and progression‐free survival than sorafenib [3, 4]. Nevertheless, therapeutic responses to AB vary widely, and treatment‐related adverse events (AEs) remain a major therapeutic challenge [5]. Consequently, there is a critical need to identify reliable biomarkers capable of predicting both therapeutic efficacy and the risk of severe AEs.

A disintegrin‐like and metalloproteinase with thrombospondin type 1 motifs 13 (ADAMTS13) is a zinc‐containing metalloproteinase which cleaves multimeric von Willebrand factor (VWF) [6, 7, 8, 9, 10, 11]. ADAMTS13 is primarily produced by hepatic stellate cells [12], whereas VWF is synthesized by vascular endothelial cells and released into the circulation as unusually large VWF (UL–VWF) multimers. Dysregulation of the ADAMTS13–VWF axis, characterized by reduced ADAMTS13 activity (ADAMTS13:AC) relative to elevated VWF antigen (VWF:Ag) levels, results in the accumulation of UL–VWF multimers, thereby promoting platelet aggregation and microvascular thrombosis [12, 13]. This imbalance becomes more pronounced with the progression of liver cirrhosis and HCC [6, 7, 8, 10, 11]. Accordingly, ADAMTS13:AC and VWF:Ag have been proposed as potential biomarkers for early HCC detection and predictors of therapeutic response to sorafenib [7, 8].

Angiogenesis plays a central role in HCC progression [14]. Emerging evidence suggests that VWF and ADAMTS13 are involved not only in thrombosis but also in angiogenesis [15], and that ADAMTS13–VWF axis dysregulation is associated with prognosis in various malignancies, including those with HCC receiving systemic therapy [6, 8, 16]. Nevertheless, the clinical relevance of this axis in patients receiving AB therapy remains unclear.

Bevacizumab, a monoclonal antibody targeting vascular endothelial growth factor (VEGF), suppresses tumor angiogenesis [17] and may influence endothelial function and coagulation pathways [18, 19]. In parallel, accumulating evidence suggests that the ADAMTS13–VWF axis is involved in immune regulation. Activation of vascular endothelial cells by tumor‐derived factors can enhance VWF release, which could suppress CD8^+^ T‐cell activity [20], whereas ADAMTS13 may modulate regulatory T‐cell (Treg) infiltration [21]. Given that AB therapy exerts antitumor effects through both immune‐mediated and antiangiogenic mechanisms, biomarkers reflecting endothelial activation—such as VWF and ADAMTS13—may be associated with both therapeutic efficacy and treatment‐related toxicity. Although VWF has been proposed as a predictive biomarker in patients with malignant melanoma receiving immune checkpoint inhibitors (ICIs) [16], evidence regarding the predictive value of VWF:Ag and ADAMTS13:AC in patients with HCC receiving AB therapy remains limited.

Thus, this study aimed to investigate the clinical significance of baseline plasma VWF:Ag and ADAMTS13:AC levels in patients with HCC receiving AB therapy and to evaluate their associations with therapeutic efficacy assessed by Response Evaluation Criteria in Solid Tumors (RECIST) and modified RECIST (mRECIST) criteria [22], liver function parameters, and the incidence of severe AEs.

## 2 Methods

### 2.1 Patients

A total of 66 patients with advanced HCC who received AB therapy between October 2020 and July 2023 in our hospital were reviewed. HCC diagnosis was established using dynamic computed tomography and/or dynamic magnetic resonance imaging in accordance with consensus‐based clinical practice guidelines of the Japan Society of Hepatology [23]. Patients aged ≥ 20 years with a tumor burden exceeding up‐to‐seven criteria were included [24, 25]. Previously, transarterial chemoembolization (TACE) was thought to be more effective than systemic therapy for patients meeting the up‐to‐seven criteria [24]. Consequently, TACE has become a standard treatment at our institution for such patients. Patients without blood samples were excluded. After applying these criteria, 58 patients with advanced HCC who received AB therapy were included (Figure 1). Based on the Barcelona Clinic Liver Cancer staging system, 34 patients were classified as stage B and 24 as stage C. All patients received AB therapy at 3‐week intervals. AB therapy was administered to 40 patients as first‐line treatment, 14 as second‐line, and four as third‐line or later treatment. AB therapy continued until the development of progressive disease (PD) or unmanageable AEs. Therapeutic efficacy was evaluated according to RECIST version 1.1 and mRECIST [22]. Based on the best overall response, patients were classified as responders (complete [CR] or partial response [PR]) or nonresponders (stable disease [SD] or PD). Safety was assessed using Common Terminology Criteria for AEs version 5.0 [26]. Patients were stratified according to AE severity into severe (Grade ≥ 3) and nonsevere (Grade 0–2, including no AEs) groups. This study was approved by the Ethics Committee of Nara Medical University and conducted in accordance with the tenets of the Declaration of Helsinki. Written informed consent was obtained from all participants before enrollment.

**FIGURE 1:**
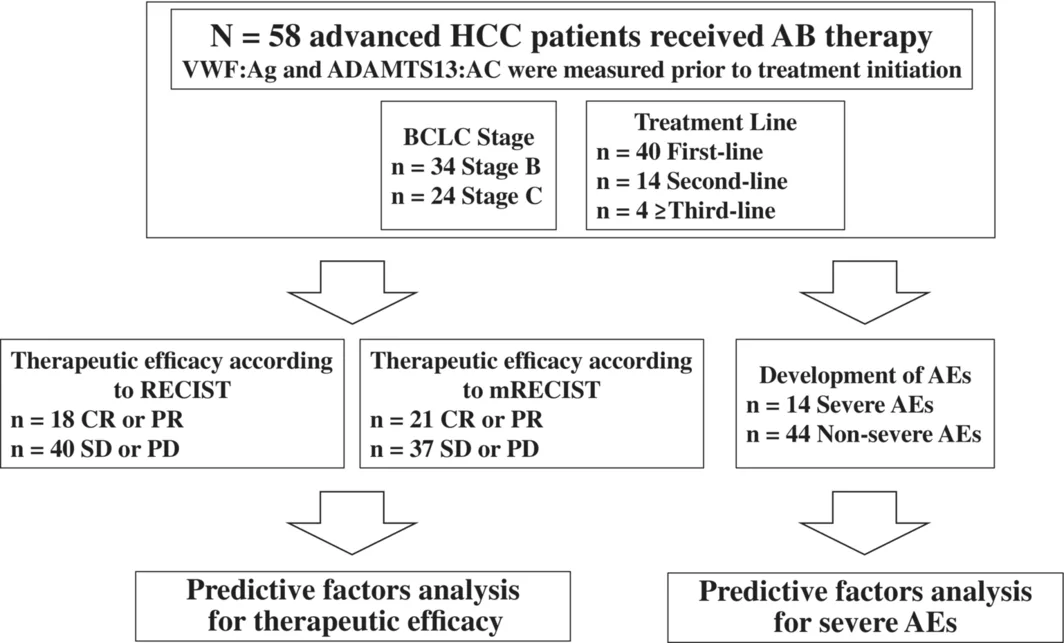
Study design. Factors predicting the therapeutic efficacy of AB therapy were examined followed by predictors of severe AEs during AB therapy. Abbreviations: AB, Atezolizumab in combination with bevacizumab; AEs, adverse events; VWF:Ag, von Willebrand factor antigen; ADAMTS13:AC, a disintegrin‐like and metalloproteinase with thrombospondin type 1 motifs 13 activity; BCLC, Barcelona Clinic Liver Cancer; RECIST, Response Evaluation Criteria in Solid Tumors; mRECIST, modified RECIST.

### 2.2 Plasma VWF Antigen and ADAMTS13 Activity Assays

Peripheral blood samples were collected from the patients before initiation of AB therapy. Blood samples were collected in plastic tubes containing one‐tenth volume of 3.8% sodium citrate as anticoagulant. Platelet‐poor plasma was prepared by centrifugation at 3000*g* for 15 min at 4°C and stored in aliquots at −80°C until analysis. Plasma VWF:Ag was measured using a rabbit polyclonal sandwich enzyme‐linked immunosorbent assay (ELISA) (Dako, Glostrup, Denmark) [27], with a reported mean VWF:Ag in healthy subjects of 102% ± 33%. Plasma ADAMTS13:AC was quantified using a sensitive chromogenic ELISA kit (Kainos Laboratories Inc., Tokyo, Japan) [28], with a reported mean activity of 99% ± 22% in healthy subjects. Measurements of VWF:Ag and ADAMTS13:AC were performed in duplicate using ELISA in accordance with the manufacturer's instructions. Intra‐ and inter‐assay coefficients of variation were < 10% and < 10% for VWF:Ag, respectively, and < 5% and < 10% for ADAMTS13:AC, respectively.

### 2.3 Statistical Analysis

All statistical analyses were performed using EZR (version 1.63; Saitama Medical Center, Jichi Medical University), a graphical user interface for R (version 4.3.1; R Foundation for Statistical Computing, Vienna, Austria). EZR is a modified version of R Commander (version 2.8‐0) that incorporates common statistical functions in biostatistical analyses [29]. Continuous variables are expressed as medians and interquartile ranges and comparisons between groups using the Mann–Whitney *U* test. Categorical variables were analyzed using Fisher's exact test. To identify predictive factors for therapeutic efficacy and the development of severe AEs during AB therapy, univariate and multivariate logistic regression analyses were performed. Variables with *p*‐values < 0.05 in univariate analysis, as well as VWF:Ag and ADAMTS13:AC, prespecified as primary candidate variables based on their established biological and clinical relevance in HCC [6, 8], were included in the multivariate model. OS was assessed using the log‐rank test. The follow‐up period was calculated from the date of initiation of AB therapy. All statistical tests were two‐tailed and a *p*‐value < 0.05 considered statistically significant.

## 3 Results

### 3.1 Patient Characteristics

Tables 1 and 2 summarize baseline clinical characteristics of the 58 patients. The median age was 74 years (interquartile range, 70–80 years), including 41 males and 17 females. In all patients, the underlying etiologies of liver disease were hepatitis B virus infection (*n* = 11), hepatitis C virus infection (*n* = 13), alcohol‐related liver disease (*n* = 13), metabolic dysfunction‐associated steatohepatitis (*n* = 12), primary biliary cholangitis (*n* = 1), and other causes (*n* = 8). Based on RECIST, 18 patients achieved CR or PR, whereas 40 patients had SD or PD. According to mRECIST, 21 patients achieved CR or PR, and 37 had SD or PD. During AB therapy, 14 patients developed severe AEs, whereas 44 patients did not. AEs were as follows: nine Grade 1 events (elevated aspartate aminotransferase (AST)/alanine aminotransferase (ALT) [*n* = 5], elevated creatinine [*n* = 1], proteinuria [*n* = 1], colitis [*n* = 1], and adrenal insufficiency with proteinuria [*n* = 1]); seven Grade 2 events (elevated AST/ALT [*n* = 3], hyper/hypothyroidism [*n* = 3], and proteinuria [*n* = 1]); 12 Grade 3 events (elevated AST/ALT [*n* = 3], elevated creatinine [*n* = 2], proteinuria [*n* = 2], colitis [*n* = 2], adrenal insufficiency [*n* = 1], pulmonary fibrosis [*n* = 1], and hypertension [*n* = 1]); and two Grade 4 events (myasthenia gravis [*n* = 1] and portal vein thrombosis [*n* = 1]). Regarding therapeutic efficacy, RECIST responders (CR/PR) had significantly higher serum albumin (Alb) levels and lower albumin–bilirubin (ALBI) scores than nonresponders (SD/PD). According to mRECIST, responders also exhibited significantly higher Alb levels and prothrombin time percentage, as well as significantly lower AST levels and ALBI scores, compared with nonresponders. In addition, the proportion of patients classified as Child–Pugh Class A was significantly higher in responders than in nonresponders. In terms of safety, des‐γ‐carboxy prothrombin (DCP) levels were significantly lower in the severe AE group than in the nonsevere AE group.

**TABLE 1:** Patient characteristics.

Characteristics	Total (*n* = 58)	RECIST		*p* *	mRECIST		*p* **
		SD/PD (*n* = 40)	CR/PR (*n* = 18)		SD/PD (*n* = 37)	CR/PR (*n* = 21)	
Age (years)	74 (70–80)	73 (69–79)	76 (72–79)	0.355	73 (67–79)	75 (72–80)	0.257
Sex (male/female)	41/17	28/12	13/5	1.000	25/12	16/5	0.560
Etiology (HBV/HCV/alcohol/MASH/PBC/others)	11/13/13/12/1/8	9/7/10/7/1/6	2/6/3/5/0/2	0.622	9/6/9/7/1/5	2/7/4/5/0/3	0.554
White blood cell (/μL)	4800 (3800–5750)	4600 (3275–5725)	5250 (4600–6375)	0.128	4600 (3200–5700)	5200 (4600–6600)	0.075
Neutrophil cell (μ/L)	2800 (2150–3700)	2600 (1875–3600)	3350 (2600–3700)	0.099	2600 (1800–3400)	3400 (2600–3700)	0.059
Lymphocyte cell (/μL)	1100 (900–1450)	1100 (900–1425)	1200 (1000–1550)	0.341	1000 (900–1400)	1200 (1000–1600)	0.140
Platelet count (×10^4^/μL)	14.0 (9.4–18.3)	13.8 (9.1–17.1)	15.5 (10.9–21.5)	0.430	13.7 (8.3–16.5)	16.4 (11.0–21.5)	0.213
C‐reactive protein (mg/dL)	0.34 (0.12–0.82)	0.30 (0.13–0.59)	0.57 (0.13–0.88)	0.528	0.28 (0.13–0.64)	0.43 (0.11–0.85)	0.639
Albumin (g/dL)	3.8 (3.4–4.0)	3.6 (3.3–3.9)	3.9 (3.7–4.1)	< 0.05	3.6 (3.3–3.9)	3.9 (3.7–4.1)	< 0.05
Aspartate aminotransferase (U/L)	40 (28–60)	43 (30–76)	35 (28–47)	0.165	45 (31–77)	33 (28–46)	< 0.05
Alanine aminotransferase (U/L)	26 (16–42)	28 (18–47)	19 (16–34)	0.337	29 (18–50)	23 (15–32)	0.278
Total bilirubin (mg/dL)	1.1 (0.6–1.6)	1.2 (0.7–1.7)	1.0 (0.6–1.1)	0.248	1.2 (0.7–1.8)	0.9 (0.6–1.1)	0.125
Prothrombin time (%)	92 (79–98)	90 (72–96)	94 (82–108)	0.093	89 (72–96)	94 (82–108)	< 0.05
Child–Pugh score (5/6/7/8/9)	31/18/7/1/1	17/15/6/1/1	14/3/1/0/0	0.150	14/15/6/1/1	17/3/1/0/0	< 0.05
Child–Pugh Class A/B	49/9	32/8	17/1	0.249	29/8	20/1	< 0.05
ALBI score	−2.44 (−2.62, −2.04)	−2.26 (−2.57, −1.99)	−2.60 (−2.70, −2.47)	< 0.05	−2.16 (−2.56, −1.98)	−2.59 (−2.70, −2.45)	< 0.05
AFP (ng/mL)	30.2 (4.0–687.5)	30.2 (3.9–930.5)	27.4 (4.4–195.9)	0.644	47.0 (4.5–1412.5)	8.2 (3.0–132.1)	0.124
DCP (mAU/mL)	443 (70–4699)	389 (74–4342)	699 (87–5623)	0.626	366 (68–4163)	696 (171–4877)	0.502
AFP‐L3 (%)	9.1 (1.8–36.1)	12.0 (1.8–35.2)	8.3 (2.6–36.1)	0.939	12.9 (2.6–39.5)	7.2 (0.9–31.8)	0.347
Maximum tumor size (cm)	3.7 (2.1–8.0)	3.7 (2.0–8.0)	4.2 (2.4–7.2)	0.737	3.6 (2.0–8.0)	4.5 (2.4–7.2)	0.710
Median tumor number	4 (1–10)	3 (1–6)	4 (1–10)	0.727	3 (1–6)	4 (2–10)	0.485
Distant metastases (presence vs. absence)	19/39	13/27	6/12	1.000	12/25	7/14	1.000
Vascular invasion (presence vs. absence)	17/41	12/28	5/13	1.000	12/25	5/16	0.560
Treatment line (first/second/ ≥ third)	40/14/4	27/10/3	13/4/1	1.000	24/10/3	16/4/1	0.737
VEGF (pg/mL)	213 (50–408)	155 (50–408)	238 (80–349)	0.626	151 (50–431)	219 (65–310)	0.870
VWF:Ag (%)	163 (124–278)	215 (147–285)	97 (80–137)	< 0.05	192 (147–293)	124 (81–159)	< 0.05
ADAMTS13:AC (%)	55 (33–82)	61 (38–87)	49 (32–70)	0.240	64 (41–92)	38 (31–63)	< 0.05
VWF/ADAMTS13 ratio	2.8 (1.7–6.4)	2.9 (1.8–6.4)	2.5 (1.7–9.0)	0.314	3.0 (1.9–7.6)	2.1 (1.6–5.0)	0.886

*Note:* Data are expressed as median (interquartile range). *p*‐Values represent comparisons between patients with SD/PD and those with CR/PR in RECIST and mRECIST.

Abbreviations: ADAMTS13:AC, a disintegrin‐like and metalloproteinase with thrombospondin type 1 motifs 13 activity; AFP, alpha‐fetoprotein; ALBI, albumin–bilirubin score; ALP‐L3, *lens culinaris* agglutinin‐reactive alpha‐fetoprotein; CR, complete response; DCP, des‐γ‐carboxy prothrombin; HBV, hepatitis B virus; HCV, hepatitis C virus; MASH, metabolic dysfunction‐associated steatohepatitis; mRECIST, modified RECIST; NS, not significant; PBC, primary biliary cholangitis; PD, progressive disease; PR, partial response; RECIST, Response Evaluation Criteria in Solid Tumors; SD, stable disease; VEGF, vascular endothelial growth factor; VWF/ADAMTS13 ratio, ratio of VWF:Ag to ADAMTS13:AC; VWF:Ag, von Willebrand factor antigen.

* SD/PD versus CR/PR.

** SD/PD versus CR/PR.

**TABLE 2:** Characteristics of patients with and without severe adverse events.

Characteristics	Nonsevere adverse events group (*n* = 44)	Severe adverse events group (*n* = 14)	*p* *
Age (years)	75 (70–80)	72 (70–78)	0.554
Sex (male/female)	31/13	10/4	1.000
Etiology (HBV/HCV/alcohol/MASH/PBC/others)	8/9/11/9/0/7	3/4/2/3/1/1	0.555
White blood cell (/μL)	4600 (3800–5900)	4950 (3475–5550)	0.723
Neutrophil cell (μ/L)	2800 (2175–3725)	2750 (1950–3500)	0.611
Lymphocyte cell (/μL)	1100 (900–1450)	1050 (925–1425)	0.935
Platelet count (×10^4^/μL)	14.3 (9.6–19.2)	12.6 (7.6–16.8)	0.403
C‐reactive protein (mg/dL)	0.40 (0.17–0.85)	0.13 (0.07–0.59)	0.110
Albumin level (g/dL)	3.8 (3.4–4.1)	3.8 (3.4–3.9)	0.505
Aspartate aminotransferase (U/L)	33 (26–43)	44 (30–76)	0.093
Alanine aminotransferase (U/L)	30 (18–47)	18 (15–20)	0.060
Total bilirubin (mg/dL)	1.1 (0.7–1.6)	0.9 (0.6–1.3)	0.259
Prothrombin time (%)	93 (81–100)	89 (76–95)	0.287
Child–Pugh score (5/6/7/8/9)	24/13/6/0/1	7/5/1/1/0	0.522
Child–Pugh Class A/B	37/7	12/2	1.000
ALBI score	−2.45 (−2.62, −2.05)	−2.43 (−2.67, −2.14)	0.971
AFP (ng/mL)	57.8 (4.4–930.5)	13.3 (3.3–36.9)	0.154
DCP (mAU/mL)	622 (108–6519)	98 (16–613)	< 0.05
AFP‐L3 (%)	10.4 (2.3–44.6)	8.8 (1.2–17.9)	0.384
Maximum tumor size (cm)	5.1 (2.1–8.0)	3.2 (2.4–4.6)	0.331
Median tumor number	3 (1–8)	5 (1–10)	0.814
Distant metastases (presence vs. absence)	17/27	2/12	0.113
Vascular invasion (presence vs. absence)	13/31	4/10	1.000
Treatment line (first/second/ ≥ third)	32/10/2	8/4/2	0.251
VEGF (pg/mL)	285 (50–441)	151 (50–215)	0.113
VWF:Ag (%)	184 (134–293)	135 (124–160)	< 0.05
ADAMTS13:AC (%)	44 (32–81)	70 (54–99)	< 0.05
VWF/ADAMTS13 ratio	3.2 (1.8–11.6)	2.2 (1.3–2.6)	< 0.05

*Note:* Data are expressed as median (interquartile range). *p*‐Values represent comparisons between patients with nonsevere and severe adverse events.

Abbreviations: ADAMTS13:AC, a disintegrin‐like and metalloproteinase with thrombospondin type 1 motifs 13 activity; AFP, alpha‐fetoprotein; ALBI score, albumin–bilirubin score; ALP‐L3%, *lens culinaris* agglutinin‐reactive alpha‐fetoprotein; DCP, des‐γ‐carboxy prothrombin; HBV, hepatitis B virus; HCV, hepatitis C virus; MASH, metabolic dysfunction‐associated steatohepatitis; PBC, primary biliary cholangitis; VEGF, vascular endothelial growth factor; VWF/ADAMTS13 ratio, ratio of VWF:Ag to ADAMTS13:AC; VWF:Ag, von Willebrand factor antigen.

* Nonsevere adverse events versus severe adverse events.

### 3.2 Plasma VWF:Ag and ADAMTS13:AC Levels

According to RECIST, plasma VWF:Ag levels were significantly lower in responders than in nonresponders, whereas ADAMTS13:AC did not differ significantly between groups (Table 1). According to mRECIST criteria, both VWF:Ag and ADAMTS13:AC levels were significantly lower in responders than in nonresponders (Table 1). When stratifying by safety outcomes, VWF:Ag levels and the VWF:Ag to ADAMTS13:AC ratio (VWF/ADAMTS13 ratio) were significantly lower in the severe AE group than in the nonsevere AE group, whereas ADAMTS13:AC levels were significantly higher (Table 2).

### 3.3 Relationship Between VWF:Ag or ADAMTS13:AC and Clinical Parameters

Correlation analyses revealed that plasma VWF:Ag levels were negatively correlated with serum Alb levels and positively correlated with AST, ALT, total bilirubin, Child–Pugh score, ALBI score, VEGF, tumor number, neutrophil‐to‐lymphocyte ratio (NLR), and platelet‐to‐lymphocyte ratio (PLR; Figure 2). In contrast, ADAMTS13:AC levels were significantly, inversely correlated with both NLR and PLR (Figure 2). However, neither VWF:Ag nor ADAMTS13:AC levels showed a significant correlation with alpha‐fetoprotein or DCP.

**FIGURE 2:**
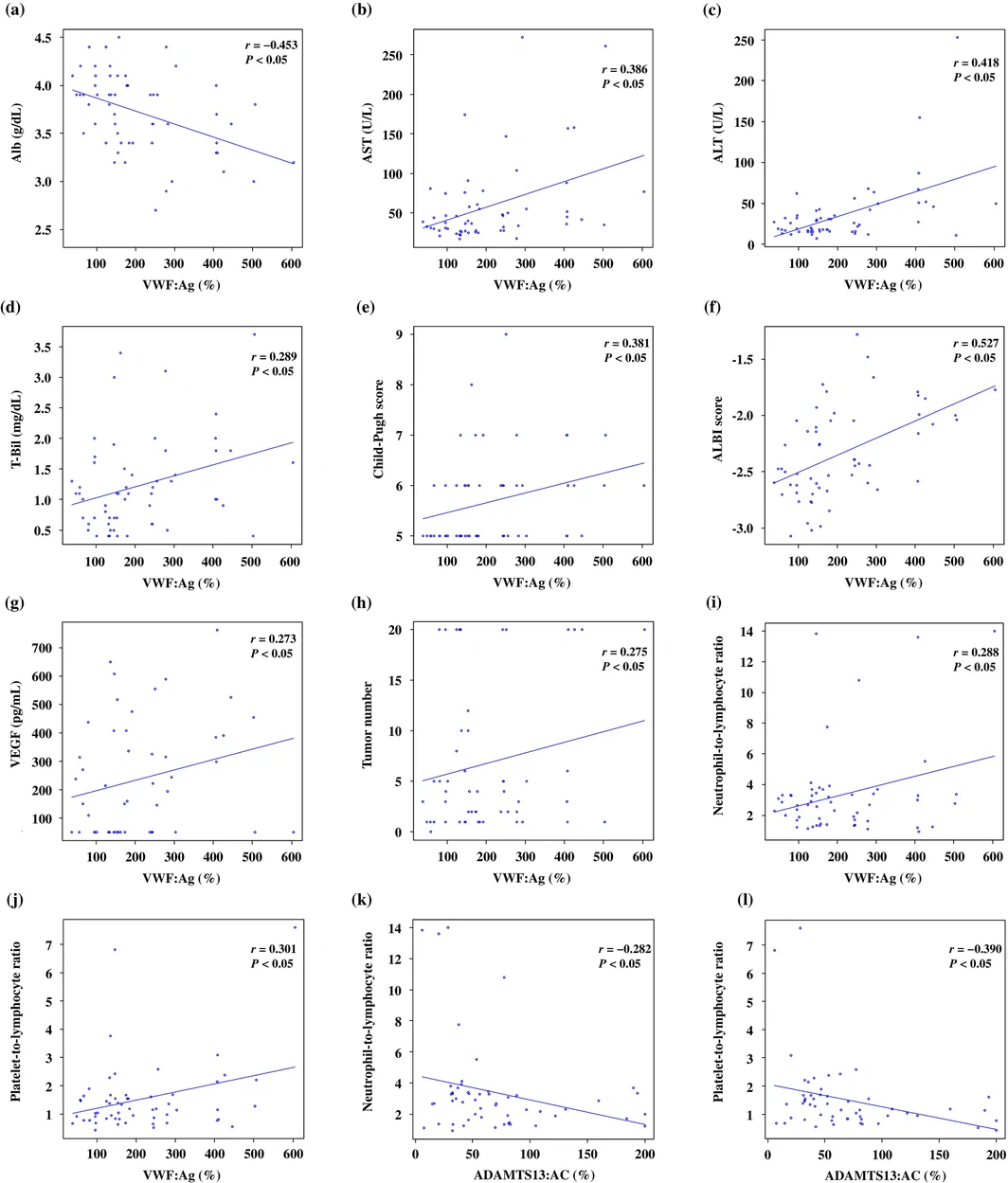
Relationship between von Willebrand factor antigen and ADAMTS13 activity with clinical parameters. Correlation analyses showing negatively correlated VWF:Ag levels with Alb levels (a) and positively correlated with AST (b), ALT (c), T‐Bil (d), Child–Pugh score (e), ALBI score (f), VEGF (g), tumor number (h), neutrophil‐to‐lymphocyte ratio (NLR) (i) and platelet‐to‐lymphocyte ratio (PLR) (j). ADAMTS13:AC levels were significantly negatively correlated with NLR (k) and PLR (l). Abbreviations: VWF:Ag, von Willebrand factor antigen; Alb, albumin; AST, aspartate aminotransferase; ALT, alanine aminotransferase; T‐Bil, total bilirubin; ALBI score, albumin–bilirubin score; VEGF, vascular endothelial growth factor; ADAMTS13:AC, a disintegrin‐like and metalloproteinase with thrombospondin type 1 motifs 13 activity.

### 3.4 Predictive Factors for AB Therapeutic Efficacy

Cut‐off values for VWF:Ag, ADAMTS13:AC, and VWF/ADAMTS13 ratio were determined using receiver operating characteristic curve analysis with the Youden index (Table S1). ALBI score and VWF:Ag were identified as significant predictors for therapeutic efficacy in univariate analyses. A multivariate analysis was performed using ALBI score, VWF:Ag, and ADAMTS13:AC, the latter implicated in HCC pathogenesis [6, 8] and identified as a potential predictor for therapeutic efficacy. Consequently, lower VWF:Ag and ADAMTS13:AC remained independent predictors in multivariate analysis according to RECIST and mRECIST criteria (Table 3).

**TABLE 3:** Association between patient characteristics and response to atezolizumab plus bevacizumab according to RECIST and mRECIST criteria.

RECIST							mRECIST					
Variable	Univariate analysis			Multivariate analysis			Univariate analysis			Multivariate analysis		
	OR	95% CI	*p*	OR	95% CI	*p*	OR	95% CI	*p*	OR	95% CI	*p*
Albumin > 4.0 g/dL	2.76	0.84–9.05	0.095				2.72	0.85–8.37	0.093			
Aspartate aminotransferase > 40 U/L	0.58	0.19–1.79	0.339				0.38	0.13–1.16	0.090			
Prothrombin time > 90%	1.25	0.41–3.82	0.695				1.41	0.48–4.14	0.534			
Child–Pugh score ≤ 6	4.25	0.49–36.9	0.189				5.52	0.64–47.6	0.120			
ALBI score > −2.60	0.29	0.09–0.95	< 0.05	0.55	0.13–2.27	< 0.05	0.30	0.95–0.97	< 0.05	0.53	0.13–2.15	0.375
Treatment line (first vs. ≥ second)	1.25	0.37–4.26	0.719				1.73	0.52–5.81	0.373			
VWF:Ag < 180%	10.10	2.04–50.4	< 0.05	9.21	1.65–51.3	< 0.05	5.25	1.47–18.8	< 0.05	5.19	1.22–22.1	< 0.05
ADAMTS13:AC > 60%	0.46	0.12–1.04	0.059	0.55	0.06–0.76	< 0.05	0.30	0.09–1.02	0.053	0.25	0.06–0.99	< 0.05
VWF/ADAMTS13 ratio < 3	2.06	0.62–6.80	0.237				1.18	0.39–3.60	0.776			

Abbreviations: ADAMTS13:AC, a disintegrin‐like and metalloproteinase with thrombospondin type 1 motifs 13 activity; ALBI score, albumin–bilirubin score; CI, confidence interval; OR, odds ratio; VWF/ADAMTS13 ratio, ratio of VWF:Ag to ADAMTS13:AC; VWF:Ag, von Willebrand factor antigen.

### 3.5 Prognosis According to VWF:Ag and ADAMTS13:AC

We compared OS based on therapeutic efficacy factors like the ALBI score, VWF:Ag, and ADAMTS13:AC, as outlined in Table 3. We also considered treatment line, distinguishing between first‐line and later second‐line or advanced options, which might influence prognosis. No significant differences in OS were observed between patients receiving first‐line treatment and those receiving second‐line or later treatment, nor between patients with a low ALBI score (≤ −2.60) and those with a high ALBI score (> −2.60). In contrast, patients with low baseline levels of VWF:Ag (< 180%) and ADAMTS13:AC (≤ 60%) exhibited significantly longer OS compared with those with high VWF:Ag (≥ 180%) or ADAMTS13:AC (> 60%; Figure 3).

**FIGURE 3:**
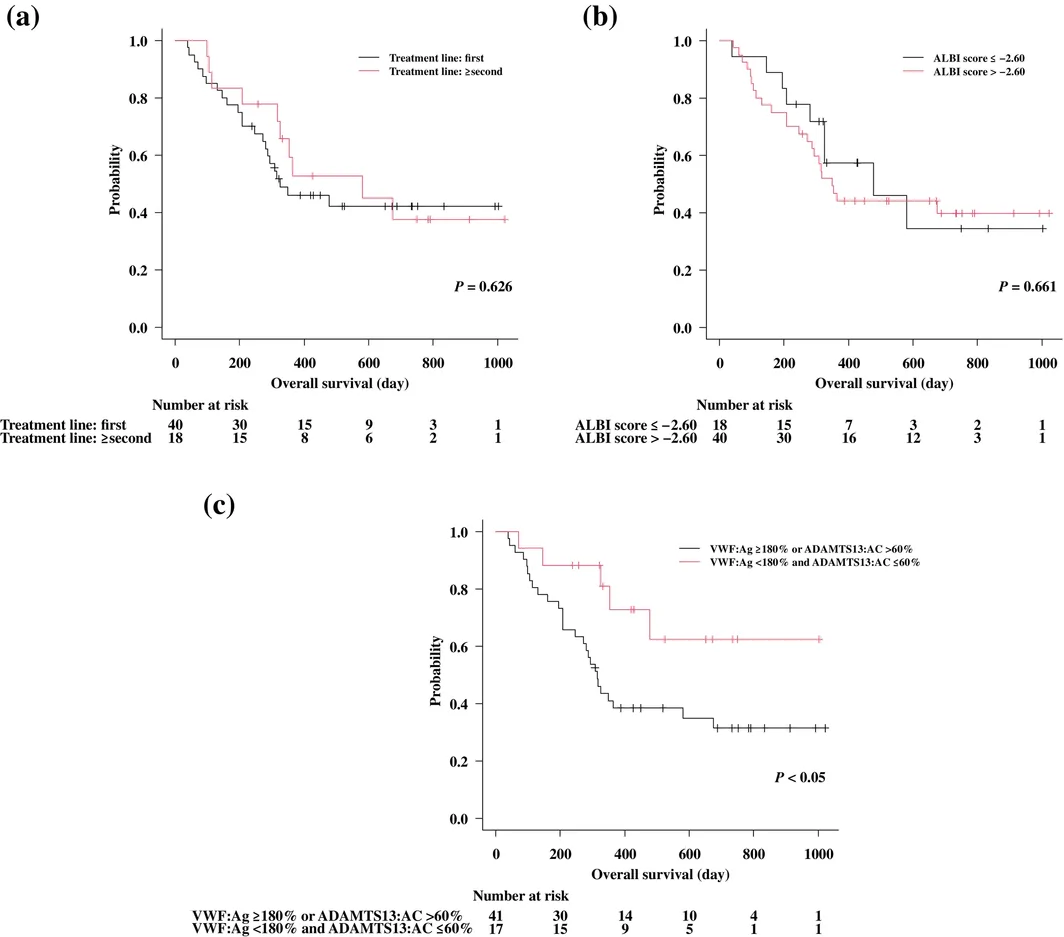
Overall survival in patients with hepatocellular carcinoma receiving atezolizumab–bevacizumab therapy. (a) No significant difference in overall survival was observed between patients receiving first‐line treatment and those receiving second‐line or later treatment. (b) No significant difference in overall survival was observed between patients with a low baseline ALBI score (≤ −2.60) and those with a high baseline ALBI score (> −2.60). (c) Patients with low baseline VWF:Ag levels (< 180%) and ADAMTS13:AC (≤ 60%) had significantly overall survival than those with high VWF:Ag (≥ 180%) or ADAMTS13:AC levels (> 60%). Abbreviations: ALBI score, albumin–bilirubin score; VWF:Ag, von Willebrand factor antigen; ADAMTS13:AC, a disintegrin‐like and metalloproteinase with thrombospondin type 1 motifs 13 activity.

### 3.6 Predictors of Severe AEs During AB Therapy

In univariate analyses, lower VWF:Ag and VWF/ADAMTS13 ratio were identified as significant predictors of severe AEs during AB therapy. VWF:Ag was excluded from the multivariate model to avoid multicollinearity with the VWF/ADAMTS13 ratio. Multivariate analysis was then performed using the VWF/ADAMTS13 ratio and DCP. DCP reflects tumor burden, microvascular invasion, and hypoxia in HCC [30, 31], reflecting advanced disease status, which can predispose patients to treatment‐related complications. Additionally, as DCP showed a strong univariate trend (*p* = 0.052), it was included in the multivariate model. Lower VWF/ADAMTS13 ratio remained a significant predictor in multivariate analysis (Table 4).

**TABLE 4:** Association between patient characteristics and severe adverse events.

Variable	Univariate analysis			Multivariate analysis		
	OR	95% CI	*p*	OR	95% CI	*p*
DCP > 200 mAU/mL	0.28	0.08–1.01	0.052	0.31	0.08–1.20	0.091
Treatment line (first vs. ≥ second)	0.54	0.16–1.87	0.332			
VWF:Ag < 180%	5.49	1.32–22.9	< 0.05			
ADAMTS13:AC > 60%	3.00	0.85–10.6	0.089			
VWF/ADAMTS13 ratio < 3	6.90	1.38–34.6	< 0.05	5.32	1.23–22.9	< 0.05

Abbreviations: ADAMTS13:AC, a disintegrin‐like and metalloproteinase with thrombospondin type 1 motifs 13 activity; CI, confidence interval; DCP, des‐γ‐carboxy prothrombin; OR, odds ratio; VWF:Ag, von Willebrand factor antigen; VWF/ADAMTS13 ratio, ratio of VWF:Ag to ADAMTS13:AC.

### 3.7 Changes in VWF:Ag and ADAMTS13:AC After AB Therapy

Plasma VWF:Ag and ADAMTS13:AC levels did not show significant changes after AB therapy (Figure S1). This pattern remained consistent when stratifying patients according to therapeutic response (CR/PR vs. SD/PD) and the incidence of severe AEs (severe AEs vs. nonsevere AEs).

## 4 Discussion

This study demonstrated the clinical importance of baseline VWF:Ag and ADAMTS13:AC levels in patients with HCC undergoing advanced‐stage therapy. Baseline VWF:Ag and ADAMTS13:AC levels were independently associated with superior therapeutic efficacy, as assessed by both RECIST and mRECIST criteria, as well as with prolonged OS. In addition, baseline VWF/ADAMTS13 ratio was identified as an independent predictor of severe AEs, highlighting the dual clinical relevance of the ADAMTS13–VWF axis in these patients.

Multivariate analyses consistently identified VWF:Ag and ADAMTS13:AC as independent predictors of therapeutic response. VWF is a well‐established biomarker of endothelial activation and vascular injury [32, 33, 34], and elevated circulating levels are frequently observed in patients with advanced liver disease and HCC [6, 7, 8, 10, 11]. Beyond its hemostatic role, growing experimental and clinical evidence indicates that VWF actively regulates angiogenesis [35]. Notably, VWF has been reported to suppress VEGF‐dependent angiogenesis through multiple intracellular and extracellular mechanisms, including interactions with integrin αvβ3 and angiopoietin‐2 [15, 36, 37]. In the present study, VWF:Ag levels showed weak‐to‐moderate positive correlations with circulating VEGF levels and tumor number. Despite the modest correlation coefficients, they suggest that VWF:Ag may partially reflect tumor burden and angiogenic activity in patients with HCC. Since bevacizumab directly targets VEGF signaling [17], baseline VWF:Ag could act as a surrogate marker of tumor angiogenic dependency and disease activity, potentially impacting both therapeutic efficacy and prognosis in patients receiving AB therapy.

Beyond its role in angiogenesis, VWF may critically influence the tumor immune microenvironment [20, 38]. Activated endothelial cells release UL–VWF multimers [6, 7, 8, 9, 10, 11], reported to suppress CD8^+^ T‐cell activity [20]. CD8^+^ T cells are key effectors of antitumor immunity elicited by ICIs, including atezolizumab [39]. Consequently, elevated VWF levels could promote an immunosuppressive tumor microenvironment, potentially reducing the therapeutic efficacy of AB. Although we did not directly evaluate tumor‐infiltrating lymphocytes or immune cell subsets in this study, patients with lower baseline VWF:Ag levels could have a more immune‐permissive environment, enabling stronger CD8^+^ T‐cell–mediated antitumor responses and improving clinical outcomes. However, as we did not measure UL–VWF multimers or CD8^+^ T‐cell activity, this interpretation remains speculative.

Additionally, an important consideration is whether VWF:Ag serves as an independent biomarker of therapeutic response or a surrogate marker for underlying hepatic functional reserve. In our analysis, VWF:Ag was significantly correlated with several indicators of liver function, including Alb, T‐Bil, and ALBI score. Indeed, VWF levels increase with the progression of liver cirrhosis and portal hypertension [11, 37, 38]. Nevertheless, in our multivariate logistic regression models adjusting for hepatic functional status, including ALBI score, lower baseline VWF:Ag remained independently associated with a favorable therapeutic response. These findings suggest that VWF:Ag may reflect tumor‐associated angiogenic or endothelial alterations beyond those attributable to hepatic function alone. However, the possibility of residual confounding due to underlying liver dysfunction cannot be completely excluded.

ADAMTS13 plays a counterregulatory role by cleaving UL–VWF multimers [6, 7, 8, 9, 10, 11], thereby modulating both thrombotic and immune‐related processes [40, 41]. Previous studies have suggested that ADAMTS13 may enhance antitumor immunity by limiting Treg infiltration within the tumor microenvironment [21]. The lower ADAMTS13:AC levels in responders may be due to increased ADAMTS13 consumption secondary to active cleavage of UL–VWF multimers in patients with heightened endothelial or immune activation. Previous studies reported similar finding [10, 42]. In line with this result, ADAMTS13:AC levels showed weak‐to‐moderate negative correlations with NLR and PLR, both reported as predictive markers for the development of AEs in patients receiving ICIs (i.e., lower NLR and PLR are associated with a higher incidence of AEs) [43, 44]. Furthermore, prior studies indicate that an increased NLR is associated with Treg expansion [44, 45]. Despite modest, these suggest that lower circulating ADAMTS13:AC levels in responders may reflect enhanced biological activity rather than diminished functional relevance. However, as we did not measure UL–VWF multimers, their cleavage products, or Treg expansion, this result remains speculative and should be interpreted with caution. Further studies are needed to elucidate the precise mechanisms underlying baseline ADAMTS13 status.

With respect to safety outcomes, baseline VWF/ADAMTS13 ratio was independently associated with severe AEs. Prior studies have reported that the development of immune‐related AEs during ICI therapy is often linked to superior therapeutic responses [46, 47, 48]. However, severe AEs in this study included both immune‐related and bevacizumab‐related events. Nonetheless, given the small number of events, subgroup analyses on these two types of AEs were not feasible, and the association between the VWF/ADAMTS13 ratio and toxicity cannot be solely attributed to immune activation.

These findings suggest intensified immune activation simultaneously contributes to both antitumor efficacy and therapy‐related toxicity. This aligns with our hypothesis that a “low VWF–high ADAMTS13” profile may increase susceptibility to heightened immune activation. This immunological milieu likely involves enhanced CD8^+^ T‐cell activity and diminished Treg function, fostering strong antitumor responses while raising the risk of severe immune‐related or systemic AEs. Together, these findings underscore the dual clinical relevance of the ADAMTS13–VWF axis in patients receiving AB therapy, supporting its potential role as a biomarker for both therapeutic efficacy and pretreatment risk stratification. Notably, baseline VWF:Ag and ADAMTS13:AC levels were not significantly altered by AB therapy. This indicates that these biomarkers are reflective of baseline host‐ and tumor‐related factors influencing therapeutic efficacy and susceptibility to severe treatment‐related AEs. Unlike conventional on‐treatment biomarkers, this feature supports their potential utility for pretreatment risk stratification and patient selection.

This study has several limitations that should be acknowledged. First, it was a single‐center study with a relatively small sample size, which may restrict the generalizability of the results. Despite conducting multivariate analyses, residual confounding cannot be entirely ruled out, highlighting the need for validation in larger, multicenter cohorts. Second, patients with advanced HCC and underlying liver cirrhosis commonly experience complications such as thrombosis and systemic inflammation, including portal vein thrombosis, bacterial overgrowth, and bacterial translocation. These conditions may influence circulating VWF:Ag and ADAMTS13:AC levels independent of tumor characteristics or treatment response [9, 49, 50], thus representing potential confounders. Third, this study focused on circulating plasma markers and did not directly assess intertumoral immune cell populations or endothelial markers. As a result, the proposed mechanistic associations between the ADAMTS13–VWF axis, angiogenesis, and immune regulation remain speculative and require further validation. Finally, given the observational nature of the study, causal relationships between the ADAMTS13–VWF axis and therapeutic outcomes cannot be established. Prospective studies incorporating serial biomarker assessments and correlative immune analyses are necessary to confirm the clinical utility of these biomarkers.

In conclusion, baseline VWF:Ag and ADAMTS13:AC levels independently predict therapeutic efficacy, whereas the VWF/ADAMTS13 ratio predicts the risk of severe AEs in patients with HCC receiving AB therapy. By reflecting the intricate interactions among tumor angiogenesis, endothelial activation, and host immune status, these biomarkers may provide clinically valuable insights for pretreatment risk stratification and personalized treatment optimization.

## Funding

The authors have nothing to report.

## Ethics Statement

This study was approved by the Human Ethics Review Committee of Nara University Hospital (protocol code: 1453; date of approval: January 13, 2017) and conducted in accordance with the Declaration of Helsinki. Data were collected with the intention of future analysis and analyzed in a retrospective manner.

## Consent

Written informed consent was obtained from all patients.

## Conflicts of Interest

The authors declare no conflicts of interest.

## Supporting information


**Table S1:** Receiver operating characteristic curve analysis of baseline biomarkers using the Youden index.
**Figure S1:** Changes in VWF:Ag and ADAMTS13:AC before and after AB therapy.

## Data Availability

The data are not publicly available due to privacy.

## References

[1] J. L. Petrick , A. A. Florio , A. Znaor , et al., “International Trends in Hepatocellular Carcinoma Incidence, 1978–2012,” International Journal of Cancer 147 (2020): 317–330. 10.1002/ijc.32723 PMC7470451 31597196

[2] K. A. McGlynn , J. L. Petrick , and H. B. El‐Serag , “Epidemiology of Hepatocellular Carcinoma,” Hepatology 73, no. Suppl 1 (2021): 4–13. 10.1002/hep.31288 PMC7577946 32319693

[3] R. S. Finn , S. Qin , M. Ikeda , et al., “Atezolizumab Plus Bevacizumab in Unresectable Hepatocellular Carcinoma,” New England Journal of Medicine 382 (2020): 1894–1905. 10.1056/NEJMoa1915745 32402160

[4] A. L. Cheng , S. Qin , M. Ikeda , et al., “Updated Efficacy and Safety Data From IMbrave150: Atezolizumab Plus Bevacizumab vs. Sorafenib for Unresectable Hepatocellular Carcinoma,” Journal of Hepatology 76 (2022): 862–873. 10.1016/j.jhep.2021.11.030 34902530

[5] S. Takaki , M. Kurosaki , N. Mori , et al., “Effects on Survival of the Adverse Event of Atezolizumab Plus Bevacizumab for Hepatocellular Carcinoma: A Multicenter Study by the Japan Red Cross Liver Study Group,” Investigational New Drugs 41 (2023): 340–349. 10.1007/s10637-023-01349-4 36995548

[6] H. Takaya , T. Namisaki , K. Moriya , et al., “Association Between ADAMTS13 Activity‐VWF Antigen Imbalance and the Therapeutic Effect of HAIC in Patients With Hepatocellular Carcinoma,” World Journal of Gastroenterology 26 (2020): 7232–7241. 10.3748/wjg.v26.i45.7232 PMC7723670 33362379

[7] H. Takaya , T. Namisaki , M. Kitade , et al., “VWF/ADAMTS13 Ratio as a Potential Biomarker for Early Detection of Hepatocellular Carcinoma,” BMC Gastroenterology 19 (2019): 167. 10.1186/s12876-019-1082-1 PMC6802329 31638892

[8] H. Takaya , T. Namisaki , N. Shimozato , et al., “ADAMTS13 and von Willebrand Factor Are Useful Biomarkers for Sorafenib Treatment Efficiency in Patients With Hepatocellular Carcinoma,” World Journal of Gastrointestinal Oncology 11 (2019): 424–435. 10.4251/wjgo.v11.i5.424 PMC6522768 31139312

[9] H. Takaya , H. Kawaratani , T. Kubo , et al., “Platelet Hyperaggregability Is Associated With Decreased ADAMTS13 Activity and Enhanced Endotoxemia in Patients With Acute Cholangitis,” Hepatology Research 48 (2018): E52–E60. 10.1111/hepr.12926 28628948

[10] H. Takaya , H. Kawaratani , Y. Tsuji , et al., “von Willebrand Factor Is a Useful Biomarker for Liver Fibrosis and Prediction of Hepatocellular Carcinoma Development in Patients With Hepatitis B and C,” United European Gastroenterology Journal 6 (2018): 1401–1409. 10.1177/2050640618779660 PMC6206538 30386613

[11] H. Takaya , M. Uemura , Y. Fujimura , et al., “ADAMTS13 Activity May Predict the Cumulative Survival of Patients With Liver Cirrhosis in Comparison With the Child‐Turcotte‐Pugh Score and the Model for End‐Stage Liver Disease Score,” Hepatology Research 42 (2012): 459–472. 10.1111/j.1872-034X.2011.00950.x 22292786

[12] M. Uemura , K. Tatsumi , M. Matsumoto , et al., “Localization of ADAMTS13 to the Stellate Cells of Human Liver,” Blood 106 (2005): 922–924. 10.1182/blood-2005-01-0152 15855280

[13] S. Okhota , I. Melnikov , Y. Avtaeva , S. Kozlov , and Z. Gabbasov , “Shear Stress‐Induced Activation of von Willebrand Factor and Cardiovascular Pathology,” International Journal of Molecular Sciences 21 (2020): 7804. 10.3390/ijms21207804 PMC7589699 33096906

[14] H. Yoshiji , S. Kuriyama , J. Yoshii , et al., “Halting the Interaction Between Vascular Endothelial Growth Factor and Its Receptors Attenuates Liver Carcinogenesis in Mice,” Hepatology 39 (2004): 1517–1524. 10.1002/hep.20218 15185292

[15] A. M. Randi , “Angiogenesis and the ADAMTS13‐VWF Balance,” Blood 130 (2017): 1–2. 10.1182/blood-2017-05-781260 28684444

[16] J. C. Stadler , L. Keller , C. Mess , et al., “Prognostic Value of von Willebrand Factor Levels in Patients With Metastatic Melanoma Treated by Immune Checkpoint Inhibitors,” Journal for Immunotherapy of Cancer 11 (2023): e006456. 10.1136/jitc-2022-006456 PMC10255197 37258039

[17] R. K. Jain , “Tumor Angiogenesis and Accessibility: Role of Vascular Endothelial Growth Factor,” Seminars in Oncology 29 (2002): 3–9. 10.1053/sonc.2002.37265 12516032

[18] K. Chen , B. Jiang , H. Yan , L. Yang , and Z. Chen , “Case Report: Bevacizumab‐Induced Cerebrovascular Events: A Case Series Report and Literature Review,” Frontiers in Oncology 15 (2025): 1395129. 10.3389/fonc.2025.1395129 PMC11847825 39995836

[19] B. C. Kuenen , M. Levi , J. C. Meijers , et al., “Analysis of Coagulation Cascade and Endothelial Cell Activation During Inhibition of Vascular Endothelial Growth Factor/Vascular Endothelial Growth Factor Receptor Pathway in Cancer Patients,” Arteriosclerosis, Thrombosis, and Vascular Biology 22 (2002): 1500–1505. 10.1161/01.atv.0000030186.66672.36 12231573

[20] A. T. Bauer , C. Gorzelanny , C. Gebhardt , K. Pantel , and S. W. Schneider , “Interplay Between Coagulation and Inflammation in Cancer: Limitations and Therapeutic Opportunities,” Cancer Treatment Reviews 102 (2022): 102322. 10.1016/j.ctrv.2021.102322 34922151

[21] D. Li , M. S. Cho , R. Gonzalez‐Delgado , et al., “The Effect of ADAMTS13 on Graft‐Versus‐Host Disease,” Journal of Cellular and Molecular Medicine 28 (2024): e18457. 10.1111/jcmm.18457 PMC11222974 38963011

[22] R. Lencioni and J. M. Llovet , “Modified RECIST (mRECIST) Assessment for Hepatocellular Carcinoma,” Seminars in Liver Disease 30 (2010): 52–60. 10.1055/s-0030-1247132 PMC12268942 20175033

[23] N. Kokudo , N. Takemura , K. Hasegawa , et al., “Clinical Practice Guidelines for Hepatocellular Carcinoma: The Japan Society of Hepatology 2017 (4th JSH‐HCC Guidelines) 2019 Update,” Hepatology Research 49 (2019): 1109–1113. 10.1111/hepr.13411 31336394

[24] M. Kudo , T. Arizumi , K. Ueshima , T. Sakurai , M. Kitano , and N. Nishida , “Subclassification of BCLC B Stage Hepatocellular Carcinoma and Treatment Strategies: Proposal of Modified Bolondi's Subclassification (Kinki Criteria),” Digestive Diseases 33 (2015): 751–758. 10.1159/000439290 26488473

[25] M. Kudo , K. Ueshima , S. Chan , et al., “Lenvatinib as an Initial Treatment in Patients With Intermediate‐Stage Hepatocellular Carcinoma Beyond Up‐to‐Seven Criteria and Child–Pugh a Liver Function: A Proof‐of‐Concept Study,” Cancers 11 (2019): 1084. 10.3390/cancers11081084 PMC6721438 31370183

[26] Japanese Translation of Common Terminology Criteria for Adverse Events (CTCAE), and Instructions and Guidelines,” International Journal of Clinical Oncology 9, no. Suppl 3 (2004): 1–82. 15818867

[27] M. Matsumoto , S. Kawaguchi , H. Ishizashi , et al., “Platelets Treated With Ticlopidine Are Less Reactive to Unusually Large von Willebrand Factor Multimers Than Are Those Treated With Aspirin Under High Shear Stress,” Pathophysiology of Haemostasis and Thrombosis 34 (2005): 35–40. 10.1159/000088546 16293984

[28] S. Kato , M. Matsumoto , T. Matsuyama , A. Isonishi , H. Hiura , and Y. Fujimura , “Novel Monoclonal Antibody‐Based Enzyme Immunoassay for Determining Plasma Levels of ADAMTS13 Activity,” Transfusion 46 (2006): 1444–1452. 10.1111/j.1537-2995.2006.00914.x 16934083

[29] Y. Kanda , “Investigation of the Freely Available Easy‐to‐Use Software ‘EZR' for Medical Statistics,” Bone Marrow Transplantation 48 (2013): 452–458. 10.1038/bmt.2012.244 PMC3590441 23208313

[30] K. Murata , H. Suzuki , H. Okano , T. Oyamada , Y. Yasuda , and A. Sakamoto , “Hypoxia‐Induced Des‐Gamma‐Carboxy Prothrombin Production in Hepatocellular Carcinoma,” International Journal of Oncology 36 (2010): 161–170. 19956845

[31] T. Kurokawa , S. Yamazaki , Y. Mitsuka , M. Moriguchi , M. Sugitani , and T. Takayama , “Prediction of Vascular Invasion in Hepatocellular Carcinoma by Next‐Generation Des‐r‐Carboxy Prothrombin,” British Journal of Cancer 114 (2016): 53–58. 10.1038/bjc.2015.423 PMC4716541 26679378

[32] A. Wibowo , R. Pranata , M. A. Lim , M. R. Akbara , and J. W. Martha , “Endotheliopathy Marked by High von Willebrand Factor (vWF) Antigen in COVID‐19 Is Associated With Poor Outcome: A Systematic Review and Meta‐Analysis,” International Journal of Infectious Diseases 117 (2022): 267–273. 10.1016/j.ijid.2021.06.051 PMC8236128 34192577

[33] L. B. Ware , M. D. Eisner , B. T. Thompson , P. E. Parsons , and M. A. Matthay , “Significance of von Willebrand Factor in Septic and Nonseptic Patients With Acute Lung Injury,” American Journal of Respiratory and Critical Care Medicine 170 (2004): 766–772. 10.1164/rccm.200310-1434OC 15201135

[34] K. Habe , H. Wada , A. Higashiyama , et al., “The Plasma Levels of ADAMTS‐13, von Willebrand Factor, VWFpp, and Fibrin‐Related Markers in Patients With Systemic Sclerosis Having Thrombosis,” Clinical and Applied Thrombosis/Hemostasis 24 (2018): 920–927. 10.1177/1076029617736382 PMC6714713 29130325

[35] C. K. Colonne , E. J. Favaloro , and L. Pasalic , “The Intriguing Connections Between von Willebrand Factor, ADAMTS13 and Cancer,” Healthcare (Basel) 10 (2022): 10. 10.3390/healthcare10030557 PMC8953111 35327035

[36] R. D. Starke , F. Ferraro , K. E. Paschalaki , et al., “Endothelial von Willebrand Factor Regulates Angiogenesis,” Blood 117 (2011): 1071–1080. 10.1182/blood-2010-01-264507 PMC3035068 21048155

[37] P. J. Lenting , C. Casari , O. D. Christophe , and C. V. Denis , “von Willebrand Factor: The Old, the New and the Unknown,” Journal of Thrombosis and Haemostasis 10 (2012): 2428–2437. 10.1111/jth.12008 23020315

[38] A. T. Bauer , J. Suckau , K. Frank , et al., “von Willebrand Factor Fibers Promote Cancer‐Associated Platelet Aggregation in Malignant Melanoma of Mice and Humans,” Blood 125 (2015): 3153–3163. 10.1182/blood-2014-08-595686 PMC4432010 25977583

[39] P. Sharma , S. Goswami , D. Raychaudhuri , et al., “Immune Checkpoint Therapy—Current Perspectives and Future Directions,” Cell 186 (2023): 1652–1669. 10.1016/j.cell.2023.03.006 37059068

[40] A. K. Chauhan , J. Kisucka , A. Brill , M. T. Walsh , F. Scheiflinger , and D. D. Wagner , “ADAMTS13: A New Link Between Thrombosis and Inflammation,” Journal of Experimental Medicine 205 (2008): 2065–2074. 10.1084/jem.20080130 PMC2526201 18695007

[41] L. Erpenbeck , M. Demers , Z. K. Zsengellér , et al., “ADAMTS13 Endopeptidase Protects Against Vascular Endothelial Growth Factor Inhibitor‐Induced Thrombotic Microangiopathy,” Journal of the American Society of Nephrology 27 (2016): 120–131. 10.1681/ASN.2014121165 PMC4696577 26038528

[42] G. C. Hugenholtz , J. Adelmeijer , J. C. Meijers , R. J. Porte , R. T. Stravitz , and T. Lisman , “An Unbalance Between von Willebrand Factor and ADAMTS13 in Acute Liver Failure: Implications for Hemostasis and Clinical Outcome,” Hepatology 58 (2013): 752–761. 10.1002/hep.26372 23468040

[43] A. Chennamadhavuni , L. Abushahin , N. Jin , C. J. Presley , and A. Manne , “Risk Factors and Biomarkers for Immune‐Related Adverse Events: A Practical Guide to Identifying High‐Risk Patients and Rechallenging Immune Checkpoint Inhibitors,” Frontiers in Immunology 13 (2022): 779691. 10.3389/fimmu.2022.779691 PMC9086893 35558065

[44] D. Michailidou , A. R. Khaki , M. P. Morelli , L. Diamantopoulos , N. Singh , and P. Grivas , “Association of Blood Biomarkers and Autoimmunity With Immune Related Adverse Events in Patients With Cancer Treated With Immune Checkpoint Inhibitors,” Scientific Reports 11 (2021): 9029. 10.1038/s41598-021-88307-3 PMC8079370 33907229

[45] H. Cheng , G. Luo , Y. Lu , et al., “The Combination of Systemic Inflammation‐Based Marker NLR and Circulating Regulatory T Cells Predicts the Prognosis of Resectable Pancreatic Cancer Patients,” Pancreatology 16 (2016): 1080–1084. 10.1016/j.pan.2016.09.007 27665172

[46] M. Qian , P. Ma , Y. Zhao , et al., “Immune Checkpoint Inhibitor Therapy in Advanced Cancer: Clinical Association of irAEs Type, Inflammatory Markers and Efficacy,” Frontiers in Immunology 16 (2025): 1662333. 10.3389/fimmu.2025.1662333 PMC12689405 41383598

[47] L. Zhang and Y. Gao , “Cutaneous Adverse Events Associated With the Efficacy and Benefit of Immune Checkpoint Inhibitors: A Systematic Review and Meta‐Analysis,” Frontiers in Oncology 15 (2025): 1689519. 10.3389/fonc.2025.1689519 PMC12634405 41278277

[48] S. Y. Hwang , M. S. Rezaee‐Zavareh , A. M. Attia , et al., “Immune‐Related Adverse Events Are Associated With Improved Outcomes After Immune Checkpoint Inhibitor Treatment in Hepatocellular Carcinoma: A Systematic Review and Meta‐Analysis,” American Journal of Gastroenterology 121 (2025): 383–395. 10.14309/ajg.0000000000003546 PMC12345381 40377239

[49] M. Pépin , A. Kleinjan , D. Hajage , et al., “ADAMTS‐13 and von Willebrand Factor Predict Venous Thromboembolism in Patients With Cancer,” Journal of Thrombosis and Haemostasis 14 (2016): 306–315. 10.1111/jth.13205 26589836

[50] B. T. Samuelson Bannow and B. A. Konkle , “Laboratory Biomarkers for Venous Thromboembolism Risk in Patients With Hematologic Malignancies: A Review,” Thrombosis Research 163 (2018): 138–145. 10.1016/j.thromres.2018.01.037 PMC5879008 29407626

